# The molecular mechanisms of autophagy-virus interactions: from antiviral defense to viral counterstrategies

**DOI:** 10.1093/femsre/fuag039

**Published:** 2026-08-17

**Authors:** Yan-mei Song, Xiao-lan Gu, Bi-feng Yuan, Xue-jie Yu

**Affiliations:** School of Public Health, Wuhan University, Wuhan 430071, China; Department of Clinical Laboratory, Gansu Provincial Hospital, Lanzhou, Gansu 730000, China; School of Public Health, Wuhan University, Wuhan 430071, China; School of Public Health, Wuhan University, Wuhan 430071, China

**Keywords:** autophagy, viral infection, antiviral immunity, viral counterstrategy, immune evasion, host-virus interaction

## Abstract

Autophagy is a highly conserved cellular degradative pathway in eukaryotes that exerts a dual regulatory role in the host antiviral defense. On the one hand, as an essential component of the host antiviral defense network, autophagy participates in the direct degradation of viral components, synergistically modulates innate immune signaling pathways including TLRs, RLRs, and cGAS-STING, and regulates adaptive immune responses such as MHC class I/II-mediated antigen presentation. On the other hand, diverse viruses have evolved sophisticated immune evasion mechanisms during long-term coevolution with the host. These viruses target key steps of autophagy, including initiation, membrane nucleation, elongation, maturation, and lysosomal fusion, to hijack or suppress the autophagic pathway and facilitate their replication. The regulation of autophagy by different viruses exhibits remarkable molecular specificity. In-depth dissection of these molecular mechanisms underlying autophagy-virus interactions will provide novel insights into the pathogenesis of viral diseases. This review systematically summarizes the core process of autophagy and its multiple regulatory mechanisms in antiviral immunity. We further highlight how viruses employ specific molecular strategies to target distinct stages of autophagy during infection, thereby hijacking the autophagic pathway to complete their replication cycles and pathogenic progression.

## Teaser

This review summarizes how autophagy acts as a key host antiviral defense by regulating immune responses, while diverse viruses have evolved specific strategies to hijack or suppress the key stages of autophagy, revealing the newly identified molecular interplay between autophagy and viruses and advancing the understanding of viral pathogenesis.

## Introduction

Viral infections pose a severe threat to human health, causing millions of deaths worldwide every year (Chen et al. 2023). Host cells defend against viral invasion through multi-layered defense mechanisms. As an evolutionarily conserved intracellular degradative pathway, autophagy plays a crucial role in antiviral immunity (Ke et al. 2022). Autophagy is a dynamic homeostatic process in which cells degrade abnormal proteins and damaged organelles via lysosomes. It is not only essential for maintaining cellular homeostasis but also critically involved in regulating innate and adaptive immunity (Wu et al. 2021). The autophagic process is coordinately mediated by autophagy-related (ATG) proteins, which regulate key steps (e.g. autophagosome formation and substrate degradation) to maintain cellular homeostasis under stress conditions such as viral infection. In antiviral defense, autophagy not only directly degrades viral particles and replication complexes, but also forms an elaborate regulatory network with the innate and adaptive immune systems. This interaction collectively maintains the integrity of the host defense system (Choi et al. 2018).

However, during long-term coevolution with their hosts, viruses have evolved diverse strategies to evade, hijack, or even exploit the autophagic pathways, turning the host homeostatic mechanism into a tool for their own replication and spread (Chiramel et al. 2013). Viruses counteract host defenses by targeting key molecules in distinct steps of autophagy, such as by inhibiting autophagosome formation, blocking autophagosome-lysosome fusion, or using autophagic membranes as replication platforms (Zhou et al. 2023). Emerging viruses represented by SARS-CoV-2 exhibit complex regulatory effects on the autophagic pathway. They can not only evade autophagy-mediated clearance but also exploit autophagy-related components to support efficient replication (Sun et al. 2023). In recent years, with the rapid development of molecular biological techniques, understanding of the molecular mechanisms underlying autophagy-virus interactions has advanced continuously. Systematic dissection of this complex interplay not only helps elucidate viral pathogenesis, but also provides an important theoretical basis for the development of novel antiviral strategies.

Although several excellent reviews have systematically described viral intervention strategies at distinct steps of autophagy, ranging from initiation to lysosomal fusion, most of these works have tended to catalog viral strategies in a stage-by-stage manner. In contrast, cross-family comparative integration and critical appraisal of contradictory findings from different experimental systems remain relatively underexplored in the current literature.

In this Review, we seek to complement descriptive enumeration with more integrated analyses in several respects. We perform cross-pathway comparisons among the TLR, RLR, and cGAS-STING axes to distill shared regulatory principles and spatial compartmentalization logic. We also critically reassess conflicting observations, such as the cell-type- and temporal-dependent discrepancies in MHC-I regulation and TLR7 signaling. In addition, we compare viral antagonistic strategies across different viruses and summarize recurring features, including stage-specific targeting, temporal dynamics, and cell-type dependence. This review summarizes the regulatory functions of autophagy in host antiviral immunity. We further highlight the molecular mechanisms by which viruses intervene in multiple key steps of the autophagic process and evade host immune surveillance to complete their replication and proliferation in host cells.

## Autophagy

### The process of autophagy

**Figure 1:**
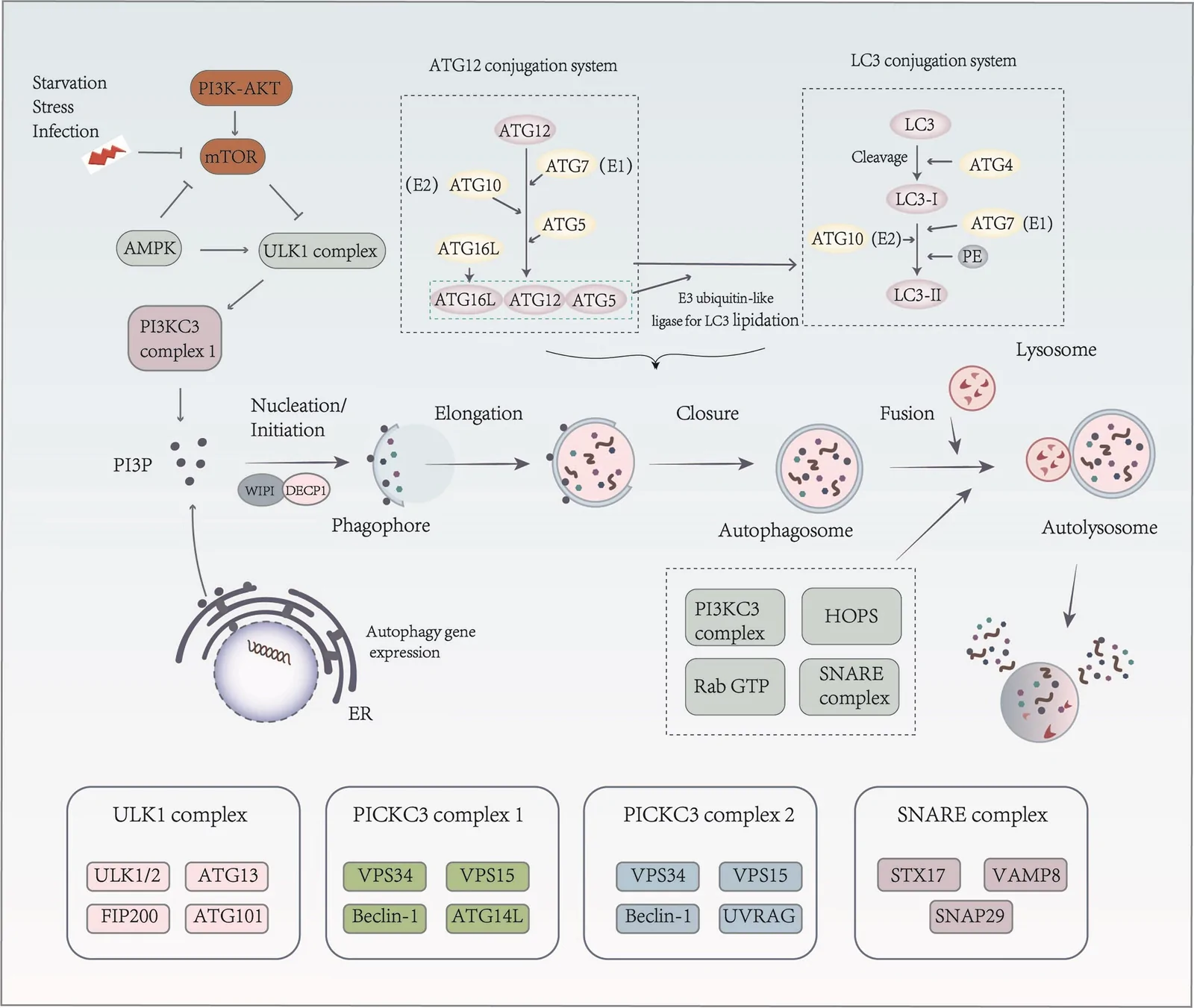
Schematic diagram of the core autophagic pathway and key molecular regulatory networks. Stress signals (e.g. starvation, infection) activate the ULK1 complex via the AMPK/PI3K-AKT-mTOR axis, which regulates PI3KC3 complex assembly and membrane-localized PI3P production to recruit downstream effectors and initiate phagophore formation. The two ubiquitin-like conjugation systems (ATG12-ATG5-ATG16 L and LC3-PE) drive membrane elongation and closure. The ATG12-ATG5-ATG16 L complex serves as an E3 ubiquitin-like ligase for LC3 lipidation. Finally, the SNARE complex, HOPS complex, and Rab GTPases mediate autophagosome-lysosome fusion, enabling substrate degradation and cellular component recycling.

Autophagy is a highly conserved catabolic process in eukaryotes. Under pathophysiological conditions such as starvation and cellular stress, intracellular components are sequestered into double-membrane autophagosomes and subsequently delivered to lysosomes for degradation. Autophagy degrades substrates such as protein aggregates, damaged organelles, and intracellular pathogens into small molecules. This process enables the recycling of cellular components and thus maintains cellular homeostasis (Levine et al. 2008, Choi et al. 2013). The canonical autophagic pathway can be divided into five key stages: initiation and nucleation, membrane elongation and expansion, autophagosome maturation, autophagosome fusion with lysosomes, and substrate degradation. The key steps of canonical autophagy are illustrated in Figure 1, and further mechanistic details are described below.

The initiation of autophagy can be triggered by multiple stress signals, including nutrient deprivation, starvation, pathogen invasion, endoplasmic reticulum stress, and hypoxia (He et al. 2009, Levine et al. 2011). Under stress conditions, the AMP-activated protein kinase (AMPK) pathway is activated and suppresses mTOR activity via phosphorylation. Meanwhile, the phosphoinositide-3-kinase-protein kinase B/Akt (PI3K-PKB/Akt) pathway is suppressed, thereby abolishing its positive regulation on mTOR (Rey-Jurado et al. 2015, Lin et al. 2017, Huang et al. 2019). Following reduced mTOR activity, the ULK1 complex is activated through dephosphorylation. This complex is composed of ULK1/2, ATG13, FIP200, and ATG101, and its activation initiates the nucleation and assembly of autophagosomes, a core step in autophagy activation (Jung et al. 2009, Zachari et al. 2017). The activated ULK1 complex translocates from the cytoplasm to the endoplasmic reticulum (ER), where it induces ER remodeling and phosphorylates the downstream class III phosphatidylinositol 3-kinase complex 1 (PI3KC3-C1) to facilitate its assembly. The core subunits of PI3KC3-C1 include VPS34, VPS15, Beclin-1, and ATG14 L (Munson et al. 2015). This complex catalyzes the generation of phosphatidylinositol 3-phosphate (PI3P), which acts as a lipid anchor to recruit downstream effectors including WIPI family proteins and DFCP1, thereby driving the formation of ER-derived autophagosomal isolation membranes (Almannai et al. 2022, Nähse et al. 2023).

Membrane elongation and maturation during autophagy depend on two ubiquitin-like conjugation systems, namely the ATG12-ATG5-ATG16 L conjugation system and the LC3-phosphatidylethanolamine (PE) conjugation system (Kocak et al. 2022). These two systems act coordinately to ensure proper elongation and closure of the autophagosomal membrane. ATG12 is initially activated by ATG7, an E1-like enzyme, and subsequently transferred to ATG10, an E2-like enzyme, before covalently binding to ATG5 to form the ATG12-ATG5 conjugate. This conjugate further interacts with ATG16 L and undergoes oligomerization to form the ATG12-ATG5-ATG16 L complex. This complex localizes mainly to the outer surface of the isolation membrane and promotes the conjugation of LC3-I to PE via its E3-like enzymatic activity, thereby driving the elongation and curvature of the isolation membrane (Nakatogawa et al. 2009, Li et al. 2019). In the LC3-PE conjugation system, LC3 is a mammalian homolog of yeast ATG8. It is initially synthesized as the precursor pro-LC3, which is cleaved by ATG4 to produce cytosolic soluble LC3-I. LC3-I is sequentially activated by ATG7 (E1-like enzyme) and ATG3 (E2-like enzyme). ATG7 forms a thioester intermediate with LC3-I and transfers it to ATG3. Finally, with the catalysis of the ATG12-ATG5-ATG16 L complex acting as an E3-like enzyme, LC3-I is covalently conjugated to PE to form LC3-II (Xie et al. 2008, Gui et al. 2019, Miller et al. 2021). LC3-II is incorporated into the autophagosomal membrane and promotes membrane elongation and closure. It serves as a hallmark for autophagosome formation, and its abundance reflects autophagic activity and the degree of autophagosome maturation.

Following autophagosome maturation, the fusion of autophagosomes with lysosomes is primarily initiated and regulated by the PI3KC3-C2 complex. This complex consists of Beclin-1, UVRAG, VPS34, and VPS15. By catalyzing the production of PI3P, it modulates membrane localization and the recruitment of downstream effector proteins. It further cooperates with the SNARE complex, HOPS complex, and Rab GTPases to mediate membrane tethering and docking, ultimately driving the fusion of autophagosomes with lysosomes to form autolysosomes (Ao et al. 2014, Wang et al. 2016). Among these components, SNARE proteins act as the core executors of membrane fusion. Key components include STX17 on autophagosomes, VAMP8 on lysosomes, and the adaptor protein SNAP29. The HOPS complex tethers autophagosomal and lysosomal membranes together and provides a platform for SNARE complex assembly. The small GTPase Rab7 functions as a molecular switch via GTP/GDP cycling and recruits the HOPS complex to trigger the fusion process (Ke et al. 2024). Autolysosomes contain a variety of acid hydrolases that degrade the sequestered substrates. The resulting small-molecule nutrients are transported back to the cytoplasm and re-enter cellular metabolic cycles (Pradel et al. 2020).

### Types of autophagy

According to the mechanisms by which cellular cargoes are delivered to lysosomes, autophagy can be classified into three main types, namely macroautophagy, microautophagy, and chaperone-mediated autophagy (Feng et al. 2014, Xiang et al. 2020). Macroautophagy involves the elongation of membrane structures that engulf cytoplasmic substrates into double-membrane vesicles known as autophagosomes. These autophagosomes ultimately fuse with lysosomes to form autolysosomes, in which the sequestered contents are degraded. The resulting macromolecules are then recycled and reused by the cell (Griffey et al. 2022). Microautophagy is characterized by the direct invagination or protrusion of the lysosomal membrane. This process sequesters cytoplasmic components destined for degradation into intraluminal vesicles, which are then degraded within the lysosome (Wang et al. 2023). Chaperone-mediated autophagy (CMA) differs from the aforementioned two pathways in that it does not rely on membrane remodeling. The cytoplasmic chaperone heat shock cognate protein 70 (HSC70) recognizes cytoplasmic protein substrates harboring the KFERQ motif and targets them to the lysosomal lumen via the lysosome-associated membrane protein 2 (LAMP2) receptor for degradation (Kaushik et al. 2018, Bourdenx et al. 2021).

Based on substrate selectivity, autophagy can be classified into selective autophagy and non-selective autophagy. Selective autophagy is mediated by autophagy receptors, which enable the specific recognition and targeted degradation of distinct substrates. Key autophagy receptors include p62/SQSTM1, NDP52, OPTN, among others. These receptors usually harbor a LC3-interacting region (LIR) motif, which mediates binding to ATG8 family proteins while enabling recognition of ubiquitinated substrates (Rogov et al. 2014, Kirkin et al. 2019). Among these, p62/SQSTM1 is a canonical selective autophagy receptor. It undergoes self-oligomerization via its PB1 domain and interacts with ATG8/LC3 family proteins through the LIR motif, thereby coordinately mediating substrate recognition and autophagosome assembly (Lamark et al. 2017). In contrast, non-selective autophagy occurs primarily under stress conditions such as nutrient deprivation, where it randomly degrades cytoplasmic components to maintain cellular energy homeostasis and intracellular stability (Nie et al. 2021).

Based on molecular mechanisms and structural features, autophagy can be further categorized into canonical autophagy and non-canonical autophagy. Canonical autophagy proceeds via the classical ATG-dependent pathway and is characterized by the formation of intact autophagosomes. In contrast, non-canonical autophagy, exemplified by LC3-associated phagocytosis (LAP), only employs a subset of the autophagy-related molecular machinery. It directly mediates LC3 lipidation on the phagosomal membrane via a Rubicon-dependent mechanism and is primarily implicated in immune regulation and pathogen clearance (Heckmann et al. 2017, 2019). LAP differs substantially from canonical autophagy in molecular mechanisms, initiating signals, and biological functions. Rather than responding to intracellular nutrient stress, its primary function is to eliminate extracellular pathogens (Heckmann et al. 2017).

## Autophagy-mediated antiviral defense Network

### Synergistic regulation between autophagy and innate immunity

As a highly conserved cellular degradation and recycling mechanism, autophagy plays a crucial regulatory role in the host antiviral innate immune response. The innate immune pathways mediated by pattern recognition receptors (PRRs) and autophagy do not function independently. Rather, they establish a synergistic defense network via multilevel and bidirectional crosstalk. Core pathways including Toll-like receptors (TLRs), RIG-I-like receptors (RLRs), and the cGAS-STING pathway can activate autophagy in response to viral invasion, thereby promoting antigen presentation and pathogen clearance. Meanwhile, autophagy precisely modulates the activities of these immune pathways through selective degradation, signaling feedback, and other mechanisms, preventing excessive inflammation and preserving immune homeostasis. Below, we systematically elaborate on the crosstalk and regulatory mechanisms between autophagy and antiviral innate immunity from the perspective of distinct innate immune pathways.

#### Crosstalk between autophagy and TLR-mediated antiviral immune pathways

TLRs are critical PRRs in innate immunity, constituting the first line of host defense against invading microbial pathogens. Based on their distinct cellular localizations, TLRs can be categorized into two subfamilies. The cell surface subfamily comprises TLR1, TLR2, TLR4, TLR5, TLR6, and TLR10, which mainly recognize extracellular pathogen components, such as bacterial cell wall components and viral surface molecules. The endosomal subfamily comprises TLR3, TLR7, TLR8, TLR9, TLR11, TLR12, and TLR13, which primarily recognize nucleic acid-based pathogen-associated molecular patterns (PAMPs). For example, TLR3 recognizes dsRNA, TLR7/TLR8 recognize ssRNA, and TLR9 recognizes CpG-rich DNA (Kawai et al. 2024). Most TLRs specifically recruit MyD88 upon recognition of PAMPs, whereas TLR3 and TLR4 primarily recruit TRIF, another TIR domain-containing adaptor molecule. These two core adaptor proteins initiate downstream signaling cascades that activate the NF-κB pathway to promote inflammatory cytokine synthesis and the IRF family to induce interferon (IFN) production. These processes collectively contribute to the innate immune response (Lee et al. 2007, Kawai et al. 2010, Balka et al. 2019).

Both TLR-mediated innate immunity and autophagy act as core host defense mechanisms against pathogen invasion, and their operations are interrelated. Recognition of PAMPs by TLRs triggers autophagy to facilitate immune responses. TLR activation and subsequent downstream signaling enhance the interaction between the core adaptor proteins MyD88/TRIF and Beclin-1, destabilize the Beclin-1-BCL-2 complex, and ultimately induce autophagy (Shi et al. 2008). Meanwhile, autophagy exerts a bidirectional regulatory role on TLR signaling, ensuring the efficiency of immune responses while preventing inflammatory damage associated with excessive TLR activation. In terms of positive regulation, autophagy can amplify TLR-mediated immune responses. Zhou et al. elucidated the mechanism of IFN-α production in plasmacytoid dendritic cells (pDCs) during HIV-1 infection. They demonstrated that both infectious and non-infectious HIV-1 particles, as well as ssRNA40, induce autophagy through TLR7 signaling, and that autophagy serves as a critical mediator of IFN-α production (Zhou et al. 2012). Consistently, Lee et al. verified that autophagy regulates TLR-dependent immune responses in pDCs. Autophagy delivers cytoplasmic ssRNA viral replication intermediates to lysosomes for detection by TLR7. In addition, ATG5 deficiency significantly diminishes TLR7-dependent interferon production upon infection with vesicular stomatitis virus or Sendai virus, indicating that autophagy is indispensable for IFN-α secretion in pDCs (Lee et al. 2007). Furthermore, ATG5 is also required for TLR9-mediated IFN-α production in Herpes simplex virus 1 (HSV-1) infected pDCs (Lee et al. 2007). However, the above conclusion that autophagy positively amplifies TLR signaling in pDCs is not universally applicable. Smith et al. reported that during influenza A virus infection of lung epithelial cells, β6 integrin drives autophagy to facilitate the targeting and lysosomal degradation of TLR7, thereby attenuating TLR7-mediated IFN-α responses(Smith et al. 2023). This discrepancy suggests that the regulatory effect of autophagy on TLR pathways is highly cell-type-dependent. Given that most existing studies have been confined to pDCs and macrophages, the regulatory principles governing the autophagy-TLR axis in mucosal epithelial cells and other critical barrier tissues remain poorly characterized. To prevent immunopathological damage caused by excessive TLR activation, autophagy also negatively regulates and terminates TLR signaling. The main mechanisms involve the selective degradation of key components in the TLR pathway. For example, autophagy targets and degrades core signaling molecules, including TRIF (the adaptor for TLR3/4) (Gentle et al. 2017, Samie et al. 2018), IKK-β (a kinase in the NF-κB pathway) (Liu et al. 2018), and p65/RelA (an NF-κB transcription factor) (Feng et al. 2017)via adaptor proteins such as p62/SQSTM1 and TAX1BP1, thereby blocking sustained signal transduction. Autophagy also mediates specific regulation through ubiquitination. Some substrates are first ubiquitinated by E3 ligases such as TRIM32, and are subsequently recognized and targeted for degradation by autophagy adaptors, highlighting the precision and specificity of this regulatory pathway (Yang et al. 2017).

#### Crosstalk between autophagy and the RLR-mediated antiviral immune pathways

RLRs including the core components RIG-I (DDX58) and MDA5 (IFIH1), are cytoplasmic pattern recognition receptors that sense viral RNA. By specifically recognizing viral RNA and initiating downstream signaling cascades, RLRs activate innate immune responses. Both receptors contain a DExD/H-box helicase domain, a C-terminal domain (CTD), and an N-terminal caspase activation and recruitment domain (CARD). Among these, the helicase domain and CTD cooperatively mediate RNA recognition (Yoneyama et al. 2024). RIG-I preferentially recognizes ssRNA or dsRNA containing 5′-diphosphate or 5′-triphosphate groups, whereas MDA5 specifically binds long dsRNA. These receptors precisely distinguish between self and non-self nucleic acids according to the distinct chemical modifications of viral and host RNAs (Brisse et al. 2019). Upon binding to viral RNA, RLRs undergo conformational changes that expose their CARD domains. RLRs then interact with MAVS through homotypic CARD-CARD interactions. MAVS is localized on mitochondria or peroxisomes. Mitochondria-localized MAVS recruits TRAF family proteins and further activates the TBK1-IKKε and IKK complexes, which in turn activate IRF3/7 and NF-κB to drive the production of type I interferons (IFN-I) and proinflammatory cytokines. Conversely, peroxisome-localized MAVS induces the expression of type III interferons or interferon-stimulated genes (ISGs) by activating IRF1. Together, these processes constitute the RLR-mediated antiviral immune defense pathway (Vazquez et al. 2015, Rehwinkel et al. 2020).

During viral infection, the RLR signaling pathway and autophagy engage in close crosstalk and form a bidirectional regulatory relationship. On the one hand, autophagy regulates the RLR signaling pathway through multiple mechanisms, exerting antiviral effects while maintaining immune homeostasis. Autophagy not only directly degrades viral nucleic acids and viral particles via the autophagosome-lysosome pathway to reduce the stimuli for RLR activation, but also selectively degrades key pathway components such as RIG-I and MAVS to prevent pathological damage caused by excessive immune activation. For instance, the E3 ubiquitin ligase RNF144B directly binds to MDA5 and catalyzes its K27/K33-linked polyubiquitination, thereby mediating the autophagy-dependent degradation of MDA5 and negatively regulating innate antiviral immune responses. Accordingly, RNF144B deficiency reduces MDA5 degradation and markedly enhances MDA5-mediated IFN-I production, thereby inhibiting the replication of encephalomyocarditis virus *in vivo* (Li et al. 2024). Recent studies have also revealed that RNF167 mediates the atypical ubiquitination and degradation of RLRs through two distinct proteolytic pathways (He et al. 2025). CCDC50 is a novel selective autophagy receptor that monitors the activation status of MDA5 and RIG-I during viral infection. By mediating the autophagic degradation of activated RIG-I/MDA5, CCDC50 inhibits excessive activation of the interferon response, thereby maintaining immune homeostasis and alleviating host tissue damage (Hou et al. 2021).

On the other hand, the RLR signaling pathway can also regulate autophagy in turn, via direct activation and indirect regulation. In direct regulation, following recognition of viral nucleic acids by RLRs, downstream MAVS signaling activates core autophagy factors such as Beclin-1 and the ATG5-ATG12 complex, directly initiating autophagy and forming an RLR-autophagy antiviral synergistic pathway (Ke et al. 2023). Studies have confirmed that upon recognition of polyI:C or Sendai virus nucleic acids, RIG-I promotes mitochondrial translocation and K63-linked polyubiquitination of Beclin-1 through the RIG-I-MAVS-TRAF6 signaling axis, thereby directly activating autophagy independently of IFN-I (Lee et al. 2018). In indirect regulation, IFN-I produced by the RLR pathway upregulates the expression of ATGs through the JAK-STAT pathway, further enhancing autophagic activity (Jin et al. 2019). Dass et al. demonstrated that IFN-I acts as a positive regulator of autophagy in macrophages infected with hHSV-2. Specifically, IFN-I activates the JAK-STAT pathway, which in turn inhibits the MAPK/ERK1/2 pathway and downregulates the activity of mTOR, a negative regulator of autophagy, ultimately promoting autophagy (Dass et al. 2024).

#### Crosstalk between autophagy and cGAS-STING antiviral immune pathways

**Figure 2:**
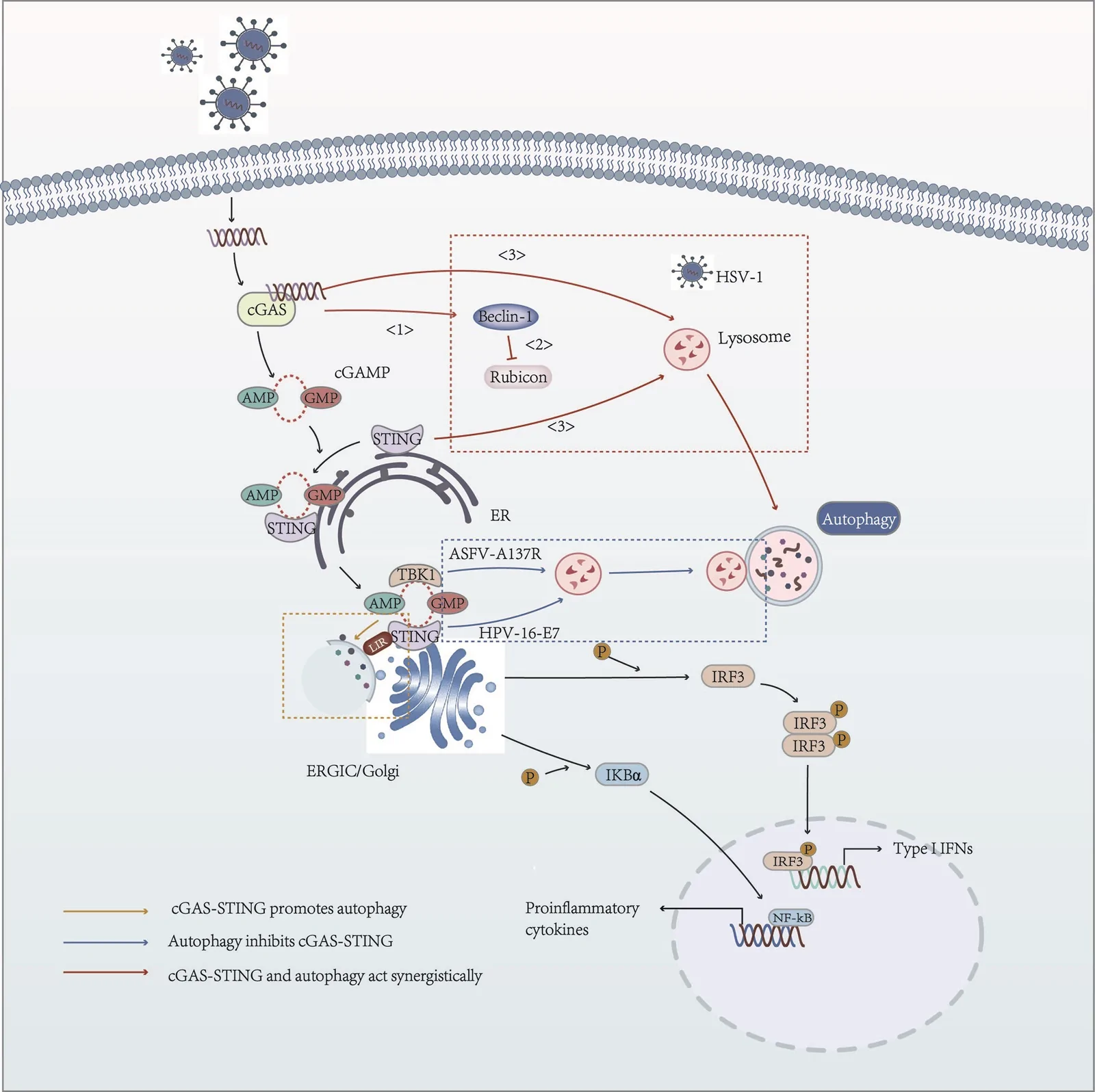
Schematic diagram of the bidirectional regulation between the cGAS-STING pathway and autophagy. Upon recognizing aberrantly localized cytoplasmic DNA, cGAS activates STING, which initiates autophagy by binding to LC3 via its LIR motif to clear pathogens. Meanwhile, autophagy degrades key components of the cGAS-STING pathway (e.g. STING, TBK1) to inhibit excessive inflammatory responses. Some viruses (e.g. HPV-16, ASFV) exploit this regulatory mechanism to degrade pathway molecules for immune evasion. The direct interaction between cGAS and Beclin-1 balances pathway activity and autophagic levels, synergistically maintaining antiviral immune homeostasis.

Aberrantly localized dsDNA in the cytoplasm serves as a core signal for the activation of the cGAS-STING pathway. The main sources of cytoplasmic dsDNA include DNA virus infection, nuclear DNA release following cellular damage, and mitochondrial DNA leakage (Hopfner et al. 2020). Upon sequence-independent recognition of cytoplasmic DNA, cGAS undergoes conformational changes and becomes activated, which in turn catalyzes the synthesis of the second messenger cyclic GMP-AMP (cGAMP) from ATP and GTP. cGAMP specifically binds to stimulator of interferon genes (STING) located on the endoplasmic reticulum (ER), inducing conformational changes in STING and its sequential translocation to the ER-Golgi intermediate compartment (ERGIC) and the Golgi apparatus. This process is mediated by COP-II-coated vesicles and the ADP-ribosylation factor (ARF) family of GTPases (Chen and Xu 2023). During its translocation between different subcellular compartments, STING orchestrates autophagy and innate immune responses via two parallel pathways. On the one hand, ERGIC-localized STING serves as a membrane platform for LC3 lipidation. By recruiting WIPI2, it promotes LC3 lipidation and triggers autophagy, thereby facilitating autophagosome formation to degrade cytoplasmic DNA and pathogens (Gui et al. 2019). On the other hand, STING translocated to the Golgi apparatus activates downstream signaling cascades and drives the expression of interferons and pro-inflammatory cytokines. STING recruits and activates TANK-binding kinase 1 (TBK1). Activated TBK1 directly phosphorylates IRF3. In parallel, TBK1 and its homolog IKKε act upstream to promote the activation of the canonical IKK complex, which then phosphorylates IκBα and the p65 subunit of NF-κB, leading to NF-κB nuclear translocation and transcriptional activation. Phosphorylated IRF3 forms homodimers and translocates into the nucleus to trigger IFN-I transcription, while IKK-dependent activation of NF-κB promotes the expression of pro-inflammatory factors (Yum et al. 2021). The cGAS-STING pathway is critical for host innate immunity against pathogens, and its interplay with autophagy coordinately modulates viral replication. This bidirectional cGAS–STING–autophagy regulatory crosstalk is illustrated in Figure 2.

In the context of viral infection, the cGAS-STING pathway exerts a crucial regulatory role in autophagy. Studies by Liu et al. have confirmed that the STING protein contains a LIR, which can directly bind to LC3 to mediate ATG5-dependent non-canonical autophagy (Liu et al. 2019). Activating signals such as cGAMP, poly(dA:dT), or HSV-1 significantly promote STING-induced autophagy. Conversely, mutational disruption of STING dimerization and cGAMP-binding capacity impairs the interaction between STING and LC3, as well as the subsequent induction of autophagy, indicating that STING activation is a key prerequisite for triggering autophagy (Liu et al. 2019). In addition, viruses can also negatively regulate the cGAS-STING pathway in a feedback manner by inducing autophagy. For example, the E7 protein encoded by human papillomavirus type 16 (HPV-16) mediates NLRX1-dependent autophagic degradation of STING, thereby promoting tumor immune escape (Luo et al. 2020). Furthermore, the A137R protein encoded by African swine fever virus (ASFV) can bind to TBK1 and mediate its lysosomal degradation, thereby inhibiting the cGAS-STING-mediated IFN-β signaling pathway (Gladue et al. 2021, Sun et al. 2022).

Notably, a bidirectional regulatory interplay exists between the cGAS-STING pathway and autophagy, and the two pathways act in concert to sustain antiviral immune responses and immune homeostasis. During HSV-1 infection, cGAS can directly interact with Beclin-1. On the one hand, this interaction inhibits cGAS activity to reduce cGAMP production and interferon generation. On the other hand, it competitively antagonizes Rubicon, a negative regulator of autophagy, thereby activating autophagy and promoting the degradation of cytoplasmic pathogenic DNA to achieve a precise balance of the innate immune response (Liang et al. 2014). Meanwhile, post-translational modifications are also involved in the fine regulation of the pathway. Trim38, an E3 ubiquitin ligase that can mediate SUMO modifications, catalyzes SUMO modifications of cGAS and STING in the early stages of uninfected or HSV-1 infections to enhance protein stability and promote pathway activation. In contrast, at the late stage of infection, Senp2 mediates the deSUMOylation of cGAS and STING, leading to their degradation via the proteasomal or CMA pathway. This process further precisely regulates pathway activity and maintains immune homeostasis (Hu et al. 2016).

#### Integrating the three axes: common principles and unresolved questions

A comparative analysis of the TLR, RLR, and cGAS-STING pathways reveals a conserved regulatory logic. In each axis, innate immune activation triggers autophagy, which subsequently modulates immune signaling. This modulation enhances pathogen clearance under physiological activation while abrogating aberrant signaling to prevent immune-mediated pathology.

Mechanistically, this is achieved through distinct yet convergent strategies. TLR pathways enlist p62 and TAX1BP1 to degrade TRIF, IKK-β, and p65. RLR pathways deploy dedicated receptors such as CCDC50 and E3 ligases including RNF144B to target activated RIG-I and MDA5. The cGAS-STING axis exploits direct receptor-autophagy protein interactions, exemplified by cGAS-Beclin-1 binding and STING-LC3 coupling. In addition, they share a unified spatial regulatory principle: signalosomes assembled on endosomes, mitochondria, and the ER-Golgi interface serve as subcellular platforms that coordinate both activation and autophagic termination in a compartment-specific manner.

Despite this conceptual framework, critical questions remain. First, the molecular switch that determines whether autophagy amplifies or terminates an immune signal is poorly defined. Emerging evidence suggests that substrate ubiquitin chain linkage patterns may govern this decision, as K63-linked chains typically facilitate signaling complex assembly whereas K27-linked chains direct autophagic degradation (Lee et al. 2018, Li et al. 2024). The balance between these modifications may therefore dictate pathway fate. Second, the degree to which the repertoire of autophagy adaptors varies across cell types, and how such diversity shapes tissue-specific immune thresholds, has not been systematically examined. Third, viral exploitation of the crosstalk between autophagy and immunity is well established. However, whether phylogenetically diverse viruses converge on shared autophagic nodes remains unresolved.

Notably, pharmacological autophagy induction restricts a broad spectrum of viruses in vitro, suggesting that harnessing endogenous defense circuits may offer a viable antiviral strategy, although the therapeutic window between immune enhancement and immunopathology warrants careful definition. Addressing these questions through cell-type-specific perturbations and quantitative in vivo flux measurements will be essential to translate mechanistic insights into therapeutic application of this self-limiting defense architecture.

### Autophagy-mediated regulation of adaptive immune responses​

Adaptive immunity represents a critical process for hosts to clear viral infections. It relies on antigen processing and presentation by antigen-presenting cells (APCs), as well as the specific activation of T and B lymphocytes. As an essential intracellular homeostatic mechanism, autophagy participates in the fine-tuning of adaptive immunity through multiple pathways. Autophagy not only regulates the presentation and cross-presentation of endogenous antigens via MHC class I molecules, but also participates in the presentation of endogenous and exogenous antigens mediated by MHC class II molecules. These regulatory events further shape the activation and effector functions of CD8⁺and CD4⁺T cells. In the following sections, we systematically elaborate on the regulatory mechanisms of autophagy in adaptive immune responses from the perspective of distinct antigen presentation pathways.

#### Regulation of MHC class I antigen presentation by autophagy​

Autophagy exerts a dual regulatory effect on the presentation of endogenous antigens mediated by MHC class I molecules. On the one hand, autophagy can suppress antiviral immune responses by modulating the internalization of MHC class I molecules. When macroautophagy is impaired in dendritic cells (DCs) (e.g. following deletion of *Atg5, Atg7*, or *Atg16L1*), LC3 lipidation is blocked, preventing the efficient recruitment of proteins such as AP2-associated kinase 1 (AAK1) to mediate MHC class I internalization. This consequently leads to the accumulation of MHC class I molecules on the cell surface, enhances CD8⁺T cell activation, and markedly promotes specific CD8⁺T cell responses against influenza A virus and lymphocytic choriomeningitis virus (Loi et al. 2016). In addition, the ORF8 protein encoded by SARS-CoV-2 can directly bind to MHC class I molecules and mediate their targeted transport to lysosomes for degradation via selective autophagy, thereby facilitating immune escape (Zhang et al. 2021). On the other hand, autophagy can also promote the antigen presentation process. Macrophages present antigens through the classical MHC class I pathway in the early stage of HSV-1 infection, while initiating the vacuolar pathway in the middle and late stages of infection, accompanied by novel autophagosomes derived from the nuclear envelope participating in antigen processing (English et al. 2009). Meanwhile, autophagy in DCs can also facilitate the processing of endogenous viral antigens (Budida et al. 2017). All the above evidence indicates that autophagy can enhance the efficiency of MHC class I molecule-mediated presentation of HSV-1 antigens. This bidirectional regulatory feature exhibits significant system-dependent discrepancies. The conclusion that autophagy mediates MHC-I internalization and degradation predominantly derives from DC models infected with IAV or coronaviruses, whereas autophagy-mediated enhancement of antigen processing in HSV-1-infected macrophages is only observed at mid-to-late stages of infection. The divergence between these conclusions is closely associated with differences in virus types, cell types, and temporal kinetics. A major limitation of current studies lies in the prevailing reliance on global knockout strategies targeting autophagy-related genes such as *Atg5* and *Atg7*. This approach makes it difficult to dissect the individual contributions of canonical macroautophagy versus other autophagy-related processes that depend on the same set of genes, for instance LC3-associated phagocytosis. Consequently, the opposing phenotypes observed across different experimental models cannot be precisely attributed to specific autophagic pathways, thereby hampering a refined understanding of the autophagy-MHC-I regulatory network (Gestal-Mato et al. 2024).

Autophagy also participates in the regulation of MHC class I-mediated cross-presentation. This process involves the processing of extracellular antigens (e.g. pathogen-derived antigens) into antigenic peptides and their subsequent presentation in endosomes or phagosomes. Notably, the MHC class I molecules required for this process are primarily derived from recycling endosomes (Blander et al. 2018). DCs are the primary cell type mediating cross-presentation, and their function is highly dependent on the vesicular transport system. Depletion of key autophagy proteins such as ATG5, ATG7, or Beclin-1 in DCs markedly impairs DC cross-presentation capacity (Cruz et al. 2017). Autophagy further regulates the long-term cross-presentation of DCs by modulating antigen storage. DCs can store antigens in endolysosomal compartments for several days to sustain MHC class I-mediated cross-presentation to CD8⁺ T cells. These compartments are closely adjacent to autophagosomes and thus susceptible to autophagic activity. Inhibition of autophagy reduces the degradation of antigen-storage compartments, thereby enhancing antigen retention (Ho et al. 2021). In addition, autophagosomes can act as antigen carriers to facilitate cross-presentation. Virus-infected donor cells secrete vesicles loaded with viral antigens into the extracellular space via the secretory autophagy pathway for delivery to immune cells. This provides a critical material basis for subsequent MHC class I-mediated cross-presentation (Uhl et al. 2009, Münz et al. 2021).

#### Regulation of MHC class II antigen presentation by autophagy

Autophagy exerts a crucial regulatory role in both endogenous and exogenous viral antigen presentation mediated by MHC class II molecules. Specifically, autophagy facilitates the processing and presentation of exogenous viral antigens by antigen–presenting cells. For example, in a model of HSV infection, DCs from *Atg5* knockout mice were unable to efficiently activate CD4⁺ T-cell responses, which ultimately led to lethality, a result that confirms the necessity of autophagy for the presentation of HSV exogenous antigens via MHC class II molecules (Lee et al. 2010). In addition, autophagy participates in MHC class II-mediated endogenous antigen presentation. During this process, cytoplasmic endogenous antigens are transported into lysosomes for degradation into antigenic peptides via CMA, dependent on the Lamp–2a–HSC70 complex located on lysosomal membranes. The antigenic peptides then bind to MHC class II molecules in lysosomal compartments to complete antigen presentation (Zhou et al. 2005). Furthermore, the autophagy receptor TAX1BP1 regulates the presentation efficiency of antigenic peptides derived from HIV and human cytomegalovirus (HCMV). TAX1BP1 maintains the stability of the invariant chain (CD74) by binding to calnexin, thereby ensuring proper trafficking of MHC class II molecules to MHC class II compartments (MIICs). This facilitates the presentation of high-affinity antigenic peptides to virus-specific CD4⁺ T cells, indicating that TAX1BP1 plays a critical role in MHC class II-restricted endogenous antigen presentation (Sarango et al. 2022).

## Viral countermeasures against the autophagic antiviral system​

### Viral intervention in autophagy initiation to promote viral replication

Viruses can activate the cellular autophagic pathway to acquire energy sources and metabolic intermediates such as ATP, amino acids, and lipids that are essential for their replication. They can also exploit the autophagosomal membrane to construct viral replication scaffolds, thereby enhancing viral replication efficiency (Gheitasi et al. 2024, Münz et al. 2025). The main pathways by which viruses regulate cellular autophagy involve inhibiting the mTOR signaling pathway, activating the AMPK signaling pathway, or directly activating the ULK1 complex to initiate autophagy (Zachari et al. 2017).

Hepatitis C virus (HCV) infection triggers endoplasmic reticulum stress, which alleviates the inhibition of ULK1 by suppressing the AKT/TSC/mTORC1 pathway, thereby upregulating cellular autophagy. Meanwhile, however, HCV inhibits PRKAA activity through multiple potential pathways, leading to the downregulation of autophagy. During HCV infection, the upregulation of autophagy induced by AKT inhibition plays a dominant role and antagonizes the autophagy–suppressive effect mediated by PRKAA, ultimately leading to an overall increase in autophagic activity. Notably, inhibition of either PRKAA or AKT promotes HCV replication, although the underlying molecular mechanisms remain to be further elucidated (Huang et al. 2013). Orf virus is a major zoonotic pathogen responsible for contagious ecthyma, which exhibits a broad host range that encompasses sheep, goats, and humans (Abu Ghazaleh et al. 2023). Orf virus orchestrates complete autophagy via a dual signaling mechanism in which it suppresses the PI3K/AKT/mTOR pathway while concomitantly activating the ERK1/2/mTOR axis. This process is contingent upon the increased phosphorylation of TSC2 and the decreased phosphorylation of mTORC1. OFTu cells (primary ovine fetal turbinate cells), naturally permissive primary cells for Orf virus infection isolated from sheep, were utilized to verify this mechanism. Robust autophagosome accumulation and elevated autophagic flux were clearly observed in this cell model, confirming that Orf virus-triggered autophagy positively facilitates viral replication (Lv et al. 2023). In addition, Enteric cytopathic human orphan virus, a member of the Enterovirus genus, frequently causes severe infections in neonates and infants, with high morbidity and mortality (Grapin et al. 2023). Recent studies have demonstrated that infection with this virus upregulates LC3–II expression, increases the number of intracellular LC3 puncta, and induces autophagosome formation in a dose–dependent manner, thereby triggering cellular autophagy. Mechanistically, it reduces the phosphorylation levels of mTOR and ULK1, and upregulates the expression of VPS34 and Beclin–1 to activate the autophagic pathway. Functional assays indicate that virus–induced autophagy promotes viral replication and VP1 protein expression, whereas inhibition of autophagy markedly attenuates VP1 expression (Wu et al. 2023).

In contrast to autophagy-inducing viruses, some viruses actively inhibit the initiation of autophagy. Their primary strategy is to evade host autophagy-mediated degradation, thereby establishing a stable intracellular environment that favors viral replication. These viruses typically block autophagy initiation by activating the mTOR signaling pathway, inhibiting the AMPK signaling pathway, or directly degrading key functional components of the ULK1 complex, ultimately promoting viral replication.

In HIV–1–infected TZM–bl cells, the HIV–1 Tat protein induces pyruvate kinase M2 (PKM2) expression. Through a dual regulatory mechanism involving activation of the PI3K/AKT pathway and inhibition of the AMPK pathway, PKM2 coordinately downregulates cellular autophagic activity via the AKT/mTOR and AMPK signaling axes, which in turn promotes HIV–1 transactivation and enhances viral replication efficiency (Zhang et al. 2017). Furthermore, HIV-1 infection suppresses autophagy in DCs. The viral Env protein activates the mTOR/S6K signaling axis via the CD4 receptor, decreases the levels of the autophagosomal protein LC3, and impairs autophagy-mediated viral degradation. These processes enhance viral transmission to T cells and ultimately compromise host immune responses (Blanchet et al. 2010). Notably, in human neural progenitor cells (LUHMES) and glioblastoma cells (A172), Zika virus infection activates the mTOR pathway, thereby promoting viral protein expression and progeny virion production. Specifically, during the early stage of Zika virus infection, autophagy activation restricts viral protein accumulation to extremely low levels. In contrast, during the late stage of infection, activated mTOR inactivates ULK1, which is the catalytic subunit of the core autophagy initiation complex, through inhibitory phosphorylation. This modification effectively blocks autophagy initiation and ultimately facilitates viral replication (Sahoo et al. 2020). Kaposi’s sarcoma–associated herpesvirus (KSHV) is the etiological agent of Kaposi’s sarcoma. The viral G protein–coupled receptor (vGPCR) encoded by KSHV, a key mediator of KS pathogenesis, promotes TSC2 phosphorylation to activate the mTOR pathway and negatively regulate autophagy, thereby inducing malignant transformation of endothelial cells (Sodhi et al. 2006). Studies have demonstrated that VDR agonists inhibit the PI3K/AKT/mTOR axis and induce autophagy via a VDR-dependent mechanism, thereby exerting anti-KS activity (Suares et al. 2019).

In summary, viral regulation of autophagy initiation is characterized by both virus species-specific and temporal features. Most RNA viruses preferentially activate autophagy through the mTOR pathway to support the metabolic and energetic demands of viral replication. In contrast, herpesviruses and retroviruses tend to suppress autophagy initiation to evade host degradation via the autophagic machinery. This differential regulation reflects the adaptive utilization of host autophagy signaling networks across distinct viral life cycles.

### Viral modulation of autophagosome nucleation to promote viral replication

Autophagosome nucleation represents a core step downstream of ULK1 complex activation, taking place mainly at membranous compartments such as the endoplasmic reticulum. Briefly, activated ULK1 mediates the assembly of the class III PI3K nucleation complex, which consists of Beclin-1, VPS34, VPS15, and ATG14. This complex catalyzes the phosphorylation of phosphatidylinositol to generate PI3P, which subsequently recruits downstream factors including ATG18 and WIPI1/2, thereby initiating phagophore formation (Chen et al. 2025). Viruses can target key components of this complex or modulate Beclin-1 activity, thereby influencing viral replication efficiency. They employ two distinct strategies. They may accelerate the assembly of the nucleation complex and promote autophagosome nucleation to provide membrane scaffolds for viral replication. They may also inhibit complex assembly and block nucleation to evade degradation by host cells.

Some viruses promote autophagosome nucleation to accelerate phagophore formation, thereby providing membrane scaffolds and metabolic resources for their own replication. Studies have demonstrated that SFTSV can regulate host canonical autophagy. Its nucleoprotein (NP) disrupts the Beclin-1-BCL2 interaction and relieves the inhibitory effect of BCL2 on Beclin-1, thereby activating Beclin-1 and initiating canonical autophagy via the RB1CC1/FIP200-Beclin-1-ATG5 axis. This autophagic response does not exert antiviral defense functions. Instead, it is hijacked by SFTSV. The virus relies on autophagosomes derived from the ERGIC and the Golgi apparatus to complete its assembly, followed by release via exocytosis mediated by autophagic vesicles, thereby facilitating the progression of its entire life cycle (Yan et al. 2022). In addition, HCV can induce mTOR-independent autophagy via its non-structural protein NS4B. NS4B employs amino acids 1–190 at its N-terminus as the functional core and acts independently of the canonical mTOR/S6K and mTOR/4E-BP1 signaling pathways. This viral protein directly binds to the host proteins Rab5 and Vps34 to form a complex, which in turn activates the kinase activity of Vps34 and initiates autophagic vesicle nucleation and autophagosome formation. These events create a favorable microenvironment for HCV replication and progeny virion production, suggesting that NS4B is a key viral protein mediating this autophagic process (Su et al. 2011).

Some other viruses evade host-mediated degradation by halting the complete autophagic pathway, for instance by inhibiting autophagic nucleation and blocking phagophore membrane formation. The neurovirulence protein ICP34.5 of HSV-1 binds to Beclin-1 and suppresses autophagy. Mutants lacking the Beclin-1-binding domain of ICP34.5 fail to inhibit autophagy in neurons and exhibit a significant reduction in their ability to induce lethal encephalitis in mice. These findings indicate that ICP34.5 suppresses autophagy by targeting Beclin-1, thereby facilitating viral replication in neurons (Orvedahl et al. 2007). Regarding the cell-type specificity of autophagy-mediated antiviral effects, Rubio et al. demonstrated that the HSV-1 Us3 serine/threonine kinase blocks the autophagic pathway in non-neuronal cells by phosphorylating and inactivating ULK1 and Beclin-1. This mechanism counteracts the antiviral function of autophagy and thereby supports efficient viral replication (Rubio et al. 2019). Furthermore, M11, a viral Bcl-2 homolog encoded by murine gammaherpesvirus 68 (γHV68), inhibits host autophagy through interaction with Beclin-1. This inhibition promotes viral survival, whereas restoration of autophagy significantly suppresses viral replication (Su et al. 2014).

Notably, human cytomegalovirus (HCMV) encodes two functionally complementary immediate-early proteins, TRS1 and IRS1. Both factors bind Beclin-1 through their conserved N-terminal domains to block autophagosome biogenesis. This autophagy-antagonizing activity occurs independently of the PKR (EIF2AK2) pathway. Mutant viral studies further demonstrate that single knockout of TRS1 or IRS1 only partially attenuates autophagic flux, whereas dual knockout completely ablates the virus’s ability to suppress autophagy. HCMV orchestrates a refined biphasic modulation of autophagy. Early infection triggers autophagy to harvest membranes and nutrients for viral proliferation, whereas late-phase expression of TRS1/IRS1 moderately represses autophagy to prevent viral degradation. γHV68 and HSV-1 depend on robust autophagy suppression to evade host clearance and sustain replication. In stark contrast, HCMV maintains partial autophagic function rather than complete pathway shutdown, and basal autophagic activity ultimately benefits viral propagation. Thus, despite sharing the conserved Beclin-1-targeting mechanism to antagonize autophagy, the three herpesviruses differ markedly in their regulatory tactics and functional outcomes (Mouna et al. 2016).

### Viral dysregulation of autophagosome expansion and maturation to promote viral replication

The expansion and maturation stages of autophagy are critical for autophagosome formation and functional maturation, involving the elongation, closure, and stable assembly of autophagosomal membranes. This complex cascade is precisely regulated by a variety of ATG proteins. The formation of the ATG12-ATG5-ATG16 complex is a key step in the expansion and maturation stages of autophagy. As an E3 ubiquitin ligase, this complex can promote the conjugation of LC3-I with phosphatidylethanolamine (PE) (i.e. the lipidation process of LC3) and simultaneously drive the expansion of the phagophore membrane (Iriondo et al. 2023). Viruses can interfere with the expansion and maturation stages of autophagy through multiple mechanisms. These interventions target not only the ATG12-ATG5-ATG16 complex and the LC3 lipidation cascade, but also other key autophagy-related molecules. According to the distinct intervention strategies employed, viral regulation of autophagic elongation and maturation can be categorized into two modes, namely autophagy induction and autophagy inhibition.

Some viruses can induce autophagic expansion and maturation while accelerating autophagosome formation, which creates a favorable environment for viral replication. Immunity-related GTPase family M (IRGM) proteins can interact with ATG5, ATG10, and LC3, thereby promoting autophagosome formation via the positive regulation of LC3 lipidation. High-throughput interaction screening has demonstrated that IRGM is among the host proteins frequently targeted by RNA viruses. The measles virus C protein, hepatitis C virus NS3 protein, and human immunodeficiency virus NEF protein all interact with IRGM and induce autophagy via an IRGM–dependent pathway, thereby enhancing viral infectivity (Grégoire et al. 2011). In addition, HIV-1 Vpu protein antagonizes the antiviral activity of host restriction factor bone marrow stromal antigen 2 (BST2/tetherin) via an LC3C-mediated noncanonical autophagic pathway (Judith et al. 2023). ATG5 plays a critical role in this process. It specifically recognizes BST2 bound to viral particles and initiates the LC3C–associated noncanonical autophagic pathway (Judith et al. 2024). Notably, this process is independent of ATG5-ATG12 complex formation but requires the involvement of ATG5 itself. This characteristic highlights the sophisticated strategies employed by viruses to regulate autophagic pathways (Olety et al. 2021). In addition to the regulatory mechanisms described above, HIV-1 can also exploit components of the autophagy machinery during autophagosome expansion and maturation. The viral Gag protein directly interacts with lipidated LC3-II on the autophagosomal membrane and uses this membrane as a scaffold for its proteolytic processing, thereby promoting viral assembly. Notably, this hijacking strategy takes advantage of autophagic membrane structures without requiring completion of the autophagic flux(Kyei et al. 2009).

In contrast, other viruses evade the host autophagic defense by inhibiting autophagosome formation and maturation, thereby promoting viral replication and survival. During the early stage of foot-and-mouth disease virus (FMDV) infection in PK-15 cells, autophagy is transiently activated, accompanied by LC3 lipidation. Subsequently, the FMDV-encoded 3C protease degrades the ATG5-ATG12 complex, a key component for autophagosome elongation, thereby inhibiting autophagosome formation and maturation and decreasing LC3-II levels. Since the ATG5-ATG12 complex also positively regulates antiviral immune pathways, viral degradation of this complex dually antagonizes both autophagy-mediated and immune-mediated restrictions on viral replication. Functional studies have demonstrated that this autophagy-interfering mechanism significantly enhances FMDV replication (Fan et al. 2017). In addition, the protease activity of FMDV 3C protease not only degrades the ATG5-ATG12 complex but also induces the extensive degradation of host proteins, particularly those involved in the NF-κB signaling pathway, which further enhances viral immune evasion (Yi et al. 2021). Viral FLICE-like inhibitory proteins (vFLIPs) encoded by KSHV, herpesvirus saimiri, and Merkel cell polyomavirus, together with cellular cFLIP, bind to ATG3 to block the interaction between ATG3 and LC3 and subsequent LC3 lipidation. This further inhibits autophagosome formation and maturation, reduces autophagic cell death, and establishes a stable environment for viral latent replication (Lee et al. 2009).

### Viral inhibition of autophagosome-lysosome fusion to evade degradation

Autophagosome-lysosome fusion is a highly precise molecular process in eukaryotic cells that relies on the coordinated regulation of multiple protein complexes. SNARE proteins, tethering complexes, and Rab GTPases represent the core regulatory factors involved in this process (Liu et al. 2021). The integrity of this fusion process is critical for the host to eliminate intracellular viruses. Following successful autophagosome-lysosome fusion, the acidic environment and hydrolytic enzymes of lysosomes degrade the sequestered viral particles, proteins, and replication complexes, thereby restricting viral replication and spread (Ke et al. 2024). Therefore, autophagosome-lysosome fusion represents a critical step in the antiviral defense of host cells.

A variety of viruses have evolved diverse strategies to block autophagosome-lysosome fusion and induce incomplete autophagy, thereby establishing an intracellular microenvironment conducive to their replication and survival. Of note, the underlying regulatory mechanisms differ substantially among distinct viruses. HCV employs a temporally coordinated dual regulatory strategy to manipulate autophagosome maturation via two functionally antagonistic autophagy regulators, Rubicon and UVRAG. Rubicon acts as a negative modulator that impedes autophagosome-lysosome fusion, whereas UVRAG potently facilitates this fusion process. Mechanistically, HCV nonstructural protein NS4B mediates robust Rubicon upregulation in early infection. Elevated Rubicon binds the Beclin-1/PI3KC3 complex, suppresses Rab7 activation and arrests autophagosome maturation, resulting in massive autophagosome accumulation. These autophagosomal double-membrane vesicles serve as the primary membranous scaffold for HCV RNA replication. As infection proceeds to the late stage, HCV markedly upregulates UVRAG. UVRAG counteracts Rubicon’ s inhibitory effect, restores autophagosome-lysosome fusion and intact autophagic flux. Recovered autophagy sustains cellular homeostasis and promotes progeny virion egress. Although the upstream molecular trigger governing this sequential expression pattern remains poorly characterized, this staged dual regulation allows HCV to remodel host cellular metabolism to match the distinct metabolic demands of each replication phase (Wang et al. 2015). At the spatial level, HCV induces lysosomal redistribution from the perinuclear region to the cell periphery by upregulating Arl8b. This causes physical segregation between autophagosomes and lysosomes, thereby suppressing autophagic flux. Studies have demonstrated that Arl8b silencing restores autophagic flux and markedly impairs viral release (Jones-Jamtgaard et al. 2019).

Human parainfluenza virus type 3 blocks autophagosome-lysosome fusion through targeting the SNARE complex. Specifically, the N-terminal domain of its P protein can directly bind to the host SNAP29 and specifically interfere with the interaction between SNAP29 and STX17. This interference inhibits the formation of functional SNARE complexes, leading to intracellular accumulation of autophagosomes that provide a favorable niche for viral replication and ultimately facilitate viral release (Ding et al. 2014). The M2 protein of influenza A virus targets the Rab7 regulatory pathway. By binding to TBC1D5, a key regulator of autophagic fusion, M2 prevents the formation of the TBC1D5-Rab7 complex and thereby blocks autophagosome-lysosome fusion. The efficiency of this viral escape mechanism relies on the stoichiometric balance between TBC1D5 and the M2 protein in host cells (Martin-Sancho et al. 2021).

HIV-1 exploits multiple viral proteins in a coordinated manner to block autophagosome-lysosome fusion, thereby evading autophagic degradation and establishing an incomplete autophagic state conducive to viral persistence. Nef functions as a principal negative regulator of autophagy, operating through several distinct mechanisms: (i) it inhibits the transcription factor TFEB, thereby attenuating lysosomal biogenesis and the expression of autophagy-related genes (Campbell et al. 2015); (ii) it promotes the BCL2-Beclin-1 interaction in a PRKN-dependent fashion, sequestering Beclin-1 and preventing assembly of the autophagy initiation complex (Castro-Gonzalez et al. 2021); (iii) it inactivates the PI3KC3 complex II, a complex specifically required for autophagosome-lysosome fusion (Chang et al. 2019); and (iv) it interferes with STX17-mediated events, compromising SNARE complex function (Grégoire et al. 2011, Kumar et al. 2018). Collectively, these actions impair autophagic flux at the transcriptional, protein-functional, and vesicle-trafficking levels, constituting a dual blockade of the autophagy pathway. The Vpr protein possesses a bidirectional regulatory capacity: it can trigger autophagy initiation while simultaneously preventing its completion. Vpr suppresses FOXO3-dependent transcription of autophagy-related genes and promotes the degradation of SNAPIN, a key SNARE complex component, ultimately disrupting autophagosome-lysosome docking and fusion (Shi et al. 2017, Alfaisal et al. 2019). Furthermore, the HIV-1 Vpu protein hijacks the V-ATPase subunit ATP6V0C, an interaction that not only interferes with autophagosome-lysosome fusion but also modulates the stability and subcellular localization of the host restriction factor Tetherin, thereby cooperatively facilitating viral assembly and release (Waheed et al. 2020).

Different members of the Picornaviridae family inhibit autophagosome-lysosome fusion via multi-target regulation with distinct mechanisms. The 3C protease of coxsackievirus B3 cleaves SNAP29 and PLEKHM1 and downregulates the transcriptional level of STX17, thereby disrupting the molecular machinery underlying autophagosome-lysosome fusion at multiple levels (Mohamud et al. 2018, Tian et al. 2018). Enterovirus D68 primarily relies on its 3C protease to specifically cleave SNAP29, thereby blocking autophagosome-lysosome fusion (Corona et al. 2018). Coxsackievirus A16 inhibits autophagosome-lysosome fusion via the coordinated action of its 2C and 3C proteins, thus inducing incomplete autophagy (Shi et al. 2015). FMDV disrupts autophagosome-lysosome fusion by directly binding to Beclin-1, thereby facilitating viral survival (Gladue et al. 2012).

Furthermore, Epstein-Barr virus and KSHV, two members of the Herpesviridae family, downregulate Rab7 expression and reduce autophagosome-lysosome colocalization, thereby indirectly inhibiting autophagosome-lysosome fusion (Granato et al. 2014, 2015). Pseudorabies virus employs a dual strategy combining the induction of selective autophagy and the blockade of autophagosome-lysosome fusion. Its gM protein triggers SQSTM1/p62-dependent selective autophagy, whereas another virally encoded homologous protein degrades SNAP29 via the CASP3-SNAP29 signaling axis. This inhibits autophagosome-lysosome fusion and ultimately establishes a state of incomplete autophagy that favors viral replication (Zhou et al. 2025).

Given that this fusion step represents a critical juncture for host cells to eliminate intracellular viruses, a diverse array of viruses has evolved multifaceted escape mechanisms that operate at multiple levels and across distinct spatial compartments. These mechanisms commonly block fusion by disrupting SNARE complex assembly, impairing Rab GTPase function, or altering organelle spatial distribution, thereby inducing incomplete autophagy. Consequently, the host autophagic defense is subverted into a replication-favorable microenvironment, representing a broadly conserved immune evasion strategy among viruses.

### Integrating viral counterstrategies: common themes, critical gaps, and future directions

Taken together, the viral counterstrategies described above reveal several unifying themes as well as key unresolved questions in the field. Table 1 summarizes these viral regulatory strategies.

**Table 1:** Viral manipulation of key autophagic stages to evade host antiviral defense.

Regulation stage	Viral regulation type	Molecular mechanism and biological effect	Representative viruses	References
Autophagy initiation	Induction	Inhibits mTOR, activates AMPK/ULK1, thereby promoting replication via energy and scaffolds.	HCV, ORFV, ECHO	(Nakatogawa et al. 2009, Munson and Ganley 2015, Münz et al. 2025)
	Inhibition	Activates mTOR, inactivates ULK1, thus evading autophagic degradation.	HIV-1 (Tat/Env), ZIKV, KSHV (vGPCR)	(Rehwinkel and Gack 2020, Nie et al. 2021)
Autophagy nucleation	Promotion	Disrupts Beclin-1-BCL2 and activates nucleation to promote phagophore formation and replication scaffolds.	SFTSV (NP), HCV (NS4B)	(Rogov et al. 2014, Rubio and Mohr 2019)
	Inhibition	Binds/phosphorylates Beclin-1 to block nucleation and evade host degradation.	HSV-1 (ICP34.5/Us3), γHV68	(Sahoo et al. 2020, Sarango et al. 2022)
Autophagy expansion	Promotion	Targets IRGM to promote LC3 lipidation and utilize non-canonical autophagy, thereby enhancing autophagosome formation, infectivity and viral release.	MeV, HCV, HIV-1 (NEF/Vpu)	(Su et al. 2014, Shi et al. 2015)
	Inhibition	Degrades ATG5-ATG12, blocks LC3 lipidation, thus inhibiting autophagosome maturation.	FMDV (3C), KSHV/HVS/MCV (vFLIP)	(Su et al. 2011, Sun et al. 2022)
Autophagosome-lysosome fusion	Blockade (incomplete autophagy)	Regulates Rubicon/UVRAG, controls fusion timing to facilitate assembly.	HCV	(Uhl et al. 2009, Vazquez and Horner 2015)
		Targets SNARE complex, induces autophagosome accumulation as replication factories.	HPIV3, CVB3, EV-D68, CA16	(Xie et al. 2008, Waheed et al. 2020, Wu et al. 2023)
		Interferes with Rab7/TBC1D5, reduces colocalization and blocks degradation.	IAV, EBV, KSHV	(Yang et al. 2017, Yi et al. 2021, Wang et al. 2023)
		Inhibits initiation and blocks fusion, thus comprehensively evading autophagy.	HIV-1(Nef/Vpu)	(Wang et al. 2015, 2016)
		Induces selective autophagy and degrades SNAP29, thus benefiting viral replication.	PRV	(Yoneyama et al. 2024)

**Note**. Abbreviations for viruses (only less common ones are listed): ORFV, Orf virus; ECHO, echovirus (enteric cytopathic human orphan virus); ZIKV, Zika virus; SFTSV, severe fever with thrombocytopenia syndrome virus; γHV68, murine gammaherpesvirus 68; MeV, measles virus; HVS, Herpesvirus saimiri; MCV, Merkel cell polyomavirus; HPIV3, human parainfluenza virus type 3; CVB3, coxsackievirus B3; EV-D68, enterovirus D68; CA16, coxsackievirus A16; PRV, pseudorabies virus.

First, viruses intervene in the autophagic process in a stage-specific manner. For example, HCV and Orf virus promote autophagosome formation to acquire membranes and nutrients required for their replication (Huang et al. 2013, Lv et al. 2023). Others block autophagic nucleation by sequestering Beclin-1 to evade degradation, as exemplified by HSV-1 and HCMV (Orvedahl et al. 2007, Mouna et al. 2016). A third group, including HIV-1 and HPIV3, allows autophagosome assembly but disrupts lysosomal fusion, leading to incomplete autophagy and the accumulation of vesicles that favor viral replication (Ding et al. 2014, Castro-Gonzalez et al. 2021). This comparison reveals a common mechanistic principle. Rather than engaging in global activation or suppression of autophagy, viruses modulate specific steps of the pathway in accordance with their replicative requirements. Nevertheless, it remains to be determined whether this stage-specific targeting arises from direct viral intervention or represents an indirect consequence of infection-related cellular stress.

Second, individual viruses often employ temporally distinct strategies. For instance, HCV suppresses autophagosome-lysosome fusion during early infection to facilitate the establishment of replication complexes, but later restores autophagic flux to enable virion egress (Wang et al. 2015). Similarly, HIV-1 promotes autophagosome formation while simultaneously blocking fusion, leading to the accumulation of autophagosomal membranes that provide a structural basis for viral assembly (Kyei et al. 2009, Castro-Gonzalez et al. 2021). Such temporal regulation indicates that conventional single-time-point measurements, which yield only a static value of autophagic activity at a given moment, are insufficient to reflect the dynamic changes in autophagic flux that occur during viral infection.

Third, the cell-type specificity of viral autophagy regulation remains systematically overlooked. For example, HSV-1 targets Beclin-1 through ICP34.5 in neurons, but utilizes Us3 kinase in non-neuronal cells to achieve similar inhibition (Orvedahl et al. 2007, Rubio et al. 2019). This discrepancy likely stems from differences in basal autophagic activity and regulatory networks among distinct cell types. Nevertheless, the majority of studies continue to rely on immortalized epithelial or fibroblast cell lines, whereas primary cells that represent natural targets of viral infection remain mostly unexplored.

## Conclusion and outlook

As a core mechanism of the host antiviral defense system, autophagy forms a multilayered antiviral network by directly degrading viral components, coordinately regulating innate immune signaling pathways, and modulating adaptive immune responses. However, viruses have evolved sophisticated countermeasures to precisely manipulate the autophagic pathway by targeting its key stages, including autophagy initiation, nucleation, elongation, and maturation, as well as autophagosome–lysosome fusion, thereby facilitating viral replication. Notably, a single virus can exert distinct regulatory effects on autophagy at different stages. For instance, HCV upregulates autophagy by inhibiting the AKT/TSC/MTORC1 pathway at the initiation stage, induces mTOR-independent autophagy via NS4B at the nucleation stage, and promotes LC3 lipidation through the IRGM pathway during the elongation and maturation stages. At the autophagosome-lysosome fusion stage, HCV mediates lysosomal redistribution via Arl8b to physically segregate autophagosomes from lysosomes, thereby suppressing autophagic flux. Similarly, HIV-1 engages in a complex bidirectional interplay with the autophagic pathway. Host cells deploy selective autophagy to eliminate viral components and subsequently activate innate and adaptive immune responses, thereby establishing a robust antiviral defense. In counteraction, HIV-1 encodes multiple proteins with dual regulatory capabilities that finely tune autophagic processes. The virus exploits the early stages of autophagy to facilitate its replication, while simultaneously blocking autophagosome maturation and fusion to evade lysosomal degradation. This dynamic equilibrium between autophagy induction and inhibition represents a core mechanism enabling HIV-1 to establish persistent infection. This phenomenon fully illustrates the complexity of virus-host interactions, and the underlying molecular mechanisms warrant further investigation. Current studies mainly focus on the canonical autophagic pathway, with limited understanding of the mechanisms underlying the role of non-canonical autophagy, such as LC3-associated phagocytosis, in viral infection. In addition, the identification of virus-specific molecular targets regulating autophagy, the spatiotemporal dynamic regulation rules, and the differences in regulatory strategies among different viral families still need to be further clarified. Future studies should integrate multi-omics technologies and real-time imaging approaches to systematically dissect the molecular networks and regulatory mechanisms underlying virus-autophagy interactions, thereby laying a solid theoretical foundation for the development of novel antiviral therapeutic strategies targeting the autophagic pathway.
